# Correction: Aleissa et al. The Association between Video Game Type and Aggressive Behaviors in Saudi Youth: A Pilot Study. *Behav. Sci*. 2022, *12*, 289

**DOI:** 10.3390/bs13110937

**Published:** 2023-11-16

**Authors:** Majid A. Aleissa, Shuliweeh Alenezi, Hassan N. Saleheen, Sumayyah R. Bin Talib, Altaf H. Khan, Shatha A. Altassan, Ahmed S. Alyahya

**Affiliations:** 1 National Family Safety Program, King Abdulaziz Medical City, Ministry of National Guard Health Affairs, Riyadh 11426, Saudi Arabia; 2 Department of Pediatrics, King Abdullah Specialized Children’s Hospital, Riyadh 11426, Saudi Arabia; 3 King Abdullah International Medical Research Center, Riyadh 11481, Saudi Arabia; 4 Department of Psychiatry, College of Medicine, King Saud University, Riyadh 11451, Saudi Arabia; 5 SABIC Psychological Health Research and Applications Chair (SPHRAC), College of Medicine, King Saud University, Riyadh 12372, Saudi Arabia; 6 Department of Psychiatry, Eradah Complex for Mental Health, Riyadh 12571, Saudi Arabia

The authors would like to make the following correction to the published paper [1]. The change is as follows:

Add this sentence to “Acknowledgement” section: 

The authors are grateful to the Deanship of Scientific Research and King Saud University for funding made available through the Vice Deanship of Scientific Research Chair.

The authors state that the scientific conclusions are unaffected. This correction was approved by the Academic Editor. The original publication has also been updated.
